# A Brief Version of the Implicit Positive and Negative Affect Test (IPANAT-18)

**DOI:** 10.5334/pb.544

**Published:** 2020-09-16

**Authors:** G. P. Hernández, S. Edo, M. Quirin, T. Rovira

**Affiliations:** 1 Stress and Health Research Group (GIES). Departament de Psicologia Bàsica, Evolutiva i de l’Educació, Universitat Autònoma de Barcelona, Barcelona, ES; 2 TUM School of Management, Technical University of Munich, Munich, DE

**Keywords:** implicit affect, IPANAT, psychometric properties

## Abstract

As self-reports of affect are limited in several regards, an indirect measure of affect, the Implicit Positive and Negative Affect Test (IPANAT; Quirin, Kazén, & Kuhl, 2009) has previously been developed and adapted to more than 10 languages (Quirin et al., 2018), showing adequate reliability and validity. Based on a sample of 242 Spanish adults (111 males), we evaluate a trimmed 18 items version of the IPANAT (IPANAT-18). Item reductive procedures consisted in a random selection of the stimuli words used in the IPANAT. Psychometric properties of the IPANAT-18 were evaluated via Confirmatory Factor Analysis. In addition, correlational analyses were used to determine the relationship between the brief and the full version of the IPANAT, and with explicit measures of affect. We replicated a two-factors structure of positive affect versus negative affect and found a good fit for the IPANAT-18 model (CFI = 1; TLI = 1; RMSEA = .00; SRMR = .03). Reliability was adequate (implicit PA, α = .86; implicit NA, α = .77) and the pattern of relationships with explicit affect measures were congruent and consistent with previous findings. Differences between the mean scores of implicit affect assessed with 18 items or 36 items were statistically non-significant, and showed strong correlations (PA, *r* = .92, *p* < .01; NA, *r* = .88, *p* < .01). In sum, the IPANAT-18 showed satisfactory psychometric properties and constitutes a useful tool for economically measuring affective processes such as in experimental and economical multiple assessment (e.g., daily diary) settings.

Traditionally, psychological assessment of affective states usually relies on the individual’s own report of their feelings. However, it has been found that people do not always identify and report emotions accurately (Quirin, Kazen, & Kuhl, 2009). The latter may partly be attributed to the complexity of affective experiences, as they are comprised of different components such as situation appraisal, subjective feelings, expressive behavior, physiological responses, and action preparation (Scherer & Moors, 2019). It has been argued that these different processes occur at a pre-reflective (i.e., automatic) and a reflective (i.e., rational) level (Lieberman, 2019). Therefore, self-report methods may not fully reflect an individual’s affective experience. Hence, the importance of studying implicit (i.e., automatic) affective processes.

Implicit affective processes are in line with a dual-process view of appraisal theories of affect (Clore & Ortony, 2000). According to this view, information can be processed with reflective propositions and rules (which convey one or more appraisal values) but alternatively (or additionally) be processed in an associative way (automatically activating learned associations between representations of the stimuli and previously stored appraisal outputs) (Moors, 2013). Accordingly, the affective experience would initiate with a pre-reflective process with several simultaneous automatic processes giving rise to experience that has not (yet) been reflected on. In line with this approach of affect as information processing, implicit affect is conceptualized as the automatic activation of cognitive representations of affective experiences (Quirin et al., 2009).

Previous research has demonstrated that affective processes, even if not fully recognized, can impact human behavior (e.g., Winkielman et al., 2005), and are related to brain processes (Lane, 2008; Pessoa, 2013), and health (e.g., Quirin & Bode, 2014; Lane, 2008; Weil et al., 2019). A number of procedures have been developed for taping affective processes indirectly, such as the Implicit Association Test (IAT; Greenwald et al., 2003; see also IAT-Anxiety, Egloff, & Schmukle, 2002), the Affect Misattribution Procedure (AMP; Payne et al., 2005). However, these measures have been developed to assess individuals’ attitudes (or self-concepts) rather than affect itself, which has been led to the development of the IPANAT.

The IPANAT aim is to assess a pre-reflective (i.e., automatic) dimension of affect, and draws on the principle of affect infusion as a method to assess implicit affect. According to this principle, affect exerts an impact on evaluative processes influencing the judgments of unrelated objects. Thus, the goal of the test is to capture the automatic affective process expressed in the participants’ biased judgments. Accordingly, the IPANAT uses participants’ ratings of the degree to which six nonsense words (i.e., SAFME, VIKES, TUNBA, TALEP, BELNI, and SUKOV) sound like six mood adjectives (i.e., happy, cheerful, energetic, helpless, tense, and inhibited). Thus, the test is composed of 36-items, which are scored on a 4-point Likert scale, ranging from doesn’t fit at all to fits very well.

The IPANAT showed good psychometric properties and construct validation (Quirin et al., 2009; Quirin et al., 2018). In addition, criterion-based validity was found by research showing relationships between implicit NA and low implicit PA with slow blood pressure recovery after harassment (Brosschot et al., 2014; van der Ploeg et al., 2014), and under unconscious stress induction (van der Ploeg et al., 2019). As well as with both stress-contingent and circadian saliva cortisol, which did not occur for explicit affect (Mossink et al., 2015; Quirin, Kazén, Rohrmann, & Kuhl, 2009). An fMRI study demonstrated that implicit (IPANAT) but not explicit negative affect predicted accuracy of recognizing briefly presented anger gestures, as well as concomitant neural correlates in the fear network of the brain (Suslow et al., 2015; see also Quirin & Lane, 2012, for the necessity of considering implicit affect in the neurosciences).

Bodenschatz et al. (2018) used eye-tracking in a healthy population to demonstrate that implicit NA predicts attention towards sad faces over and above self-reported depressive symptoms. Kazén et al. (2014) found that implicit NA predicted local processing, whereas implicit PA predicted global processing in individuals with low versus high emotion regulation abilities, respectively, these effects were not found for explicit affect. Additional studies demonstrated validity of the IPANAT as an affect measure that is incremental to explicit affect (e.g., Dekker & Johnson, 2018; Quirin et al, 2011; Remmers et al., 2016). Hence, implicit affect assessed via the IPANAT appears to contribute the understanding of affective phenomena.

In addition, the IPANAT has been adapted to many languages, displaying good psychometric properties (e.g., Hernández et al., 2020; Shimoda et al., 2014; Sulejmanov & Spasovski, 2017). Results from ten different countries showed that the best-fitting model consisted of two factors corresponding to positive affect and negative affect (on average, χ^2^/df = 2.53, CFI = .96, TLI = .91). Both factors showed a good reliability coefficient, on average, implicit PA, α = .81; implicit NA, α = .78 (Quirin et al., 2018).

Investigations on affect and health often require economical assessments. For example, due to the fact that affective processes are fleeting after experimental affect induction (see Hermans et al., 2001), because sometimes participants respond to the IPANAT in multiple assessments (like in ecological momentary assessment studies), or simply because it is administered in conjunction with time consuming other measures. Therefore, the purpose of this study was to create and evaluate a brief version of the original test (called IPANAT-18 in the remainder of this article). A validated brief version of the test could also improve the reliability on some experimental designs (e.g., if there is need of repeated measures of affect), as well as avoid extra burden or boredom to participants. Thus, a brief version would improve the instrument’s utility without sacrificing its psychometric properties.

## Method

### Participants

The sample included 242 Spanish adults (111 males). Participants’ age after classification into age bands of 18–24, 25–34, 35–44, 45–54, and 55–65 was distributed as follows: 18%, 18%, 26.8%, 18.9% and 18.3%. Participants were recruited online by a Spanish market research firm (CERES), they received 12 euros as compensation for their participation. The only requirement for participation was to be above 18 years. Participants first saw a full description of the experiment, which served simultaneously as the informed consent form. Participants who provided consent were then given a URL directing them to the experiment. More than 90% (i.e., 218) of participants reported to have been born in Spain. Regarding the education level, the majority of participants self-reported to have a university degree or above (52%). Otherwise, 37% reported a high school degree, and 11% reported a secondary school degree.

### Materials

#### Implicit Affect scale

A Spanish version of the IPANAT was used (see Hernández et al., 2020). All testing took place online via Qualtrics (Qualtrics Provo, 2013). In total, the experiment took approximately 10 minutes to complete. A computerized version of the IPANAT presented one item each per screen, after the presentation of the instruction (i.e., cover story) of the IPANAT. Then, participants were asked to provide judgments of six artificial words across six mood adjectives. For each of the artificial words (*SAFME, TALEP, BELNI, SUKOV, GOLIP, and KERUS*) participants indicated on a four-point answer scale (1 = *doesn’t fit at all*, 2 = *fits somewhat*, 3 = *fits quite well*, and 4 = *fits very well*) to what extend does the sound of the artificial word convey each of the following moods: happy, helpless, energetic, tense, cheerful, and inhibited. Thus, the test consisted of 36-items. The artificial words were randomly presented to avoid order effects, each adjective within the same artificial word was also randomized, and the six mood adjective belonging to each artificial word were presented subsequently. Global scores for implicit PA and implicit NA were computed by averaging the scores derived from positively valence, and negatively valence adjectives (following Quirin, et al., 2009).

#### Explicit affect scales

After answering the IPANAT participants were presented with a series of personality and affect questionnaires used to examine construct validity of the IPANAT. Explicit PA and NA were assessed with two instruments. First, we used the broadly applied Positive and Negative Affect Schedule (PANAS, Watson et al., 1988; Spanish version: Lopez et al., 2015). Second, explicit affect was also assessed by asking participants for explicit mood judgments of the same mood adjectives included in the IPANAT (i.e., asking individuals to report the extent to which they feel happy, cheerful, energetic, helpless, tense, and inhibited at the moment) on a rating scale from 0 (not at all) to 10 (absolutely) (following Quirin et al., 2009). Analogously to the original IPANAT, we composed a PA and an NA scale computing average scores for happy, cheerful, and energetic, versus helpless, tense, and inhibited, respectively.

### Statistical Analyses

The goal of the present study was to create and evaluate a brief version of the IPANAT. As other projective tests, the IPANAT uses judgments of artificial words to track changes on responses to ambiguous stimuli with the objective of revealing pre-reflective emotions. As detailed before, the instrument items are composed of six mood adjectives that are assessed several times, then the 36 items are in fact six truly different items asked repeatedly to capture biased responses. Thus, for the brief version of the IPANAT it is paramount to identify the number of repetitions of the items and not which particular items are needed to keep in a brief version (since they are redundant), thus a random selection of the right number of items should yield similar psychometric properties that the full test. As suggested by Taber (2018), high levels of Cronbach’s alpha indicate that items in a scale elicit the same pattern of responses (which implies they are redundant), even though a higher number of items in a scale improve the reliability, additional items measuring the same thing as the existing items leads to redundancy that is inefficient, because almost no additional useful information is obtained, nonetheless the instrument takes longer to administer.

Since we aimed to improve the usefulness of the test, in our study reliability analysis for different number of items were tested via Cronbach’s alpha coefficient, to determine the best ratio between the length of the test and good internal consistency. This item reduction analysis based on classical test theory was found to be a reliable item reduction method (Erhart et al., 2010) in comparison with other methods like Rasch item-fit analysis. As suggested by Erhart et al. (2010), our study accompanied this item reduction method by additional analysis (i.e., confirmatory factor analysis) to corroborate the psychometric properties of the instrument. Once Cronbach’s alpha coefficient provided a notion of the least number of items required to keep the psychometric properties of the original IPANAT, item reductive procedure consisted of a random selection of the stimuli words used in the IPANAT. Then, the newly stablished set of items were extracted for the original 36-items. Finally, the descriptive statistics, reliability coefficient, and latent structure of the full IPANAT were compare with the brief version.

As mentioned above, the latent structure of the IPANAT-18 was evaluated using Confirmatory Factor Analysis (CFA). The CFA model tested was based on the model proposed by authors of the original test and previous findings with the IPANAT (see Quirin et al., 2018). The CFA model expressed the hypothesis that the IPANAT measures two factors, implicit NA and implicit PA. Scores for each one of the six mood adjectives assessed (i.e., 3 for PA and 3 for NA) were calculated by averaging across ratings of the combination of the mood adjective and the three artificial words, then the corresponding 3 adjectives were loaded to its belonging factor. It was a restricted model, which allowed each of the items to load on the respective predicted factor only. Previous findings indicate that a correlation between the two underlying factors could occur (see Quirin, et al., 2018). Thus, in our study the two factors were set to be non-orthogonal, to better explore this possibility. According to Izquierdo et al. (2014), to allow the covariance of the latent factors of the model is the better way to corroborate its possible orthogonality.

The CFA models included error variances for each item and were set to load with a coefficient of 1. We estimated factor loadings via diagonally weighted least squares (DWLS) estimator, which has specifically been designed for ordinal data (Cheng-Hsien, 2016). We used Chi-squared values and degrees of freedom for each model to assess the fit of the CFA models. As well as Comparative Fit Index (CFI; Bentler, 1990), the TLI (Tucker-Lewis index), the Root-Mean-Square Error of Approximation (RMSEA), and Standardized Root Mean Square Residual (SRMR), as they are commonly recommended to assess absolute measures of fit (Browne & Cudeck, 1992; Jackson et al., 2009; Steiger & Lind, 1980). Following guidelines for Hopper et al. (2008), the present study used the next thresholds for determining model fit: Chi-squared (CMIN/df) less than 3, CFI ≥ 0.95, TLI ≥ 0.95, RMSEA ≤ 0.05 and SRMR ≤ 0.08.

Finally, we used correlational analysis and Z-tests to determine the relationships between the brief and the full version of the IPANAT, and with explicit measures of affect. Basic statistical analyses were conducted using IBM SPSS Statistics 22.0. In addition, Confirmatory Factor Analysis (CFA) were performed using R 3.6 and RStudio 1.2.

## Results

**Figure 1:**
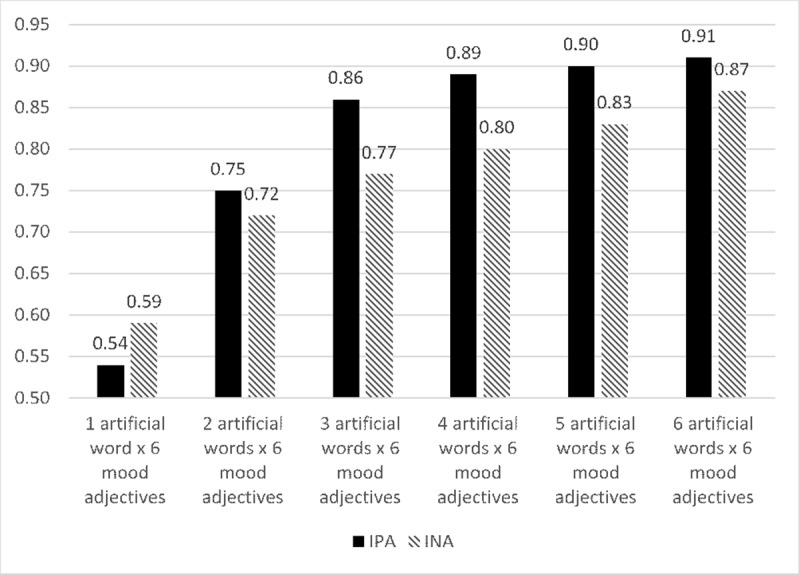
Reliability of the IPANAT’s scales showed by items reduction.

An analysis of the internal consistency of the IPANAT’s scales while performing item’s reduction (see Figure 1), determined that the best ratio between number of items and an acceptable level of alpha coefficient were three artificial words (i.e., 18 items). Since the alpha coefficients for 18 items corresponded to the least number of items with similar reliability coefficient to the ones reported for the different versions of the full IPANAT (see Quirin et al., 2018). Therefore, for the IPANAT-18 three artificial words were randomly selected (i.e., SAFME, TALEP and BELNI) from the stimuli words used in the IPANAT-S.

After having completed the test, participants responded to a question about the presumed underlying aim of the IPANAT. Twelve individuals suggested that the test might assess affective states and were excluded from the initial sample of 242 participants (4.95% of the sample). Descriptive statistics (mean scores, standard deviations, skewness, and kurtosis) for the brief and the full version of the IPANAT can be found in Table 1. There were no missing data. After evaluating the assumptions of multivariate normality and linearity, we identified that the assumption of multivariate normality is slightly violated in our sample. Therefore, we used the diagonally weighted least squares (DWLS) estimator, since this method provides more accurate parameter estimates (Mîndrilă, 2010). Regarding sample size, it was determined that the size we used in the present study is adequate for the stability of the parameter estimates, since 10 participants per estimated parameter appears to be the general consensus (see Schreiber et al., 2006). In the CFA model we specify 6 regressions, 1 covariance, and 6 variances, totalling 13 parameters that need to be estimated. Since we have a final sample size of 230, we have an acceptable ratio of 17.69 participants to 1 parameter estimated.

**Table 1:** Descriptive statistics and reliability coefficient of the brief and full version of the Implicit Positive and Negative Affect Test.

	M	*SD*	*SK*	*K*	*α*

Implicit PA (Full version)	1.82	0.58	0.20	–0.89	0.91
Implicit NA (Full version)	1.59	0.44	0.60	–0.41	0.87
Implicit PA (IPANAT-18)	1.82	0.61	0.19	–1.00	0.86
Implicit NA (IPANAT-18)	1.57	0.46	0.78	0.42	0.77

*Note*: *n* = 230.

As Table 1 shows, the mean scores for PA are higher than the mean score for implicit NA. The latter is consistent with previous findings with the IPANAT (Quirin et al., 2018). Table 1 also shows that the internal consistency estimates for the IPANAT-18 scales reached an acceptable level, implicit PA obtained an alpha coefficient of .86, while implicit NA was .77. Moreover, the alpha coefficients are comparable to the ones reported by the original version of the test (Quirin et al., 2009).

### Factor Analysis

**Figure 2:**
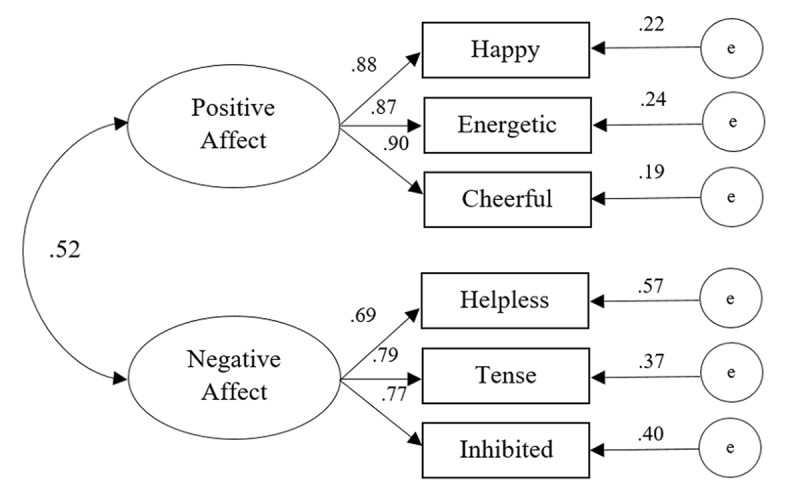
Results from Confirmatory Factor Analysis (model 2) for IPANAT-18 (*n* = 230).

The model tested for the brief version of the IPANAT-18 obtained a χ^2^ of 3.93, 8 degrees of freedom, a χ^2^/df (CMIN) of 0.49, with a CFI of 1, the TLI was also 1, the RMSEA was 0.00, while the SRMR was 0.02. According to Hu and Bentler (1999), those values indicate a good fit between the model and the observed data (see also Schreiber et al., 2006). Table 2 depicts the χ^2^ and fit indices of the full and brief version of the test, and Table 3 depicts standardized and unstandardized coefficients of the CFA Models. Along with Figure 2, the results suggest an acceptable model fit for a two-factorial solution of the IPANAT-18. Moreover, the fit indices obtained by the brief version (18-items) are slightly lower, yet comparable to fit indices found for the full version on this sample, and to the ones reported for ten different versions of the full test (see Quirin et al., 2018).

**Table 2:** Fit Indices of Models Tested in Confirmatory Factor Analysis (*n* = 230).

Model	χ^2^ *(df)*	χ^2^ */df*	CFI	TLI	RMSEA	SRMR

1. IPANAT	3.23(8)	0.40	1	1	0.00	0.02
2. IPANAT-18	3.93(8)	0.49	1	1	0.00	0.03

*Note*: 1 = Full-IPANAT (36 items), restricted bi-factorial model (Implicit Positive/Negative affect), not allowing for cross loadings between factors; 2 = IPANAT-18 (18 items), same structure than model 1; CFI = comparative fit index; TLI = Tucker-Lewis index; RMSEA = root mean square error of approximation; SRMR = standardized root mean square residual.

**Table 3:** Standardized and Unstandardized Coefficients for CFA Model 1(IPANAT) and Model 2(IPANAT-18) (*n* = 230).

Observed variable	Latent construct	IPANAT			IPANAT-18		

		*β*	*B*	*SE*	*β*	*B*	*SE*

Happy (Feliz)	PA	0.94	1.00		0.88	1.00	
Energetic (Activo)	PA	0.92	1.03	0.07	0.87	0.94	0.08
Cheerful (Alegre)	PA	0.89	0.97	0.07	0.90	0.99	0.09
Helpless (Desamparado)	NA	0.75	1.00		0.68	1.00	
Tense (Tenso)	NA	0.83	1.23	0.11	0.79	1.24	0.16
Inhibited (Inhibido)	NA	0.85	1.26	0.11	0.77	1.22	0.16

### Relationships between scales of the brief and full versions of the IPANAT

The differences between the mean scores of implicit affect assessed with 18 items or 36 items were statistically non-significant. For example, differences for implicit PA brief and full version was *t*(229) = –.35, *p* > .05, and for implicit NA *t*(229) = 1.22, *p* > .05. In addition, implicit affect mean scores assessed with the 18-items and 36-items versions showed strong correlations (implicit PA, r = .92; implicit NA, r = .88).

### Relationships between IPANAT-18 and explicit scales of affect

As shown in Table 4, the correlations between the IPANAT-18 and explicit affect measures are of moderate strength. In addition, Z-tests were run to compare the correlations between implicit and explicit scales of affect. For implicit negative affect, the results show that the correlation with explicit negative affect (assessed by PANAS) is significantly higher than the correlation with explicit positive affect, z = 2.441, p < .01. Inversely, it was found that implicit positive affect (assessed by Same Adjectives Scale), was more strongly correlated to explicit positive affect than to explicit negative affect, z = 3.107, p < .01.

**Table 4:** Pearson correlations among Implicit Affect (IPANAT-18), Explicit affect (PANAS), and Explicit scale (Same Adjectives than on IPANAT).

Measure	IPANAT-18 Implicit PA	IPANAT-18 Implicit NA

PANAS PA	0.15*	0.07 *ns*
Explicit scale PA (Same Adjectives)	0.26**	0.08 *ns*
PANAS NA	0.15*	0.29**
Explicit scale NA (Same Adjectives)	–0.05 *ns*	0.15***

*Note*: *n* = 230 ** *p* < .05 *** *p* < .01 ns = non-significant.

### Different versions of the IPANAT-18

Statistical analysis were also performed on the non-selected stimuli words of the IPANAT (i.e., SUKOV, GOLIP and KERUS). Results indicated that this set of three artificial words also shows good psychometric properties. The fit indices of the CFA model of this brief version were CMIN 0.52, CFI 0.99, TLI 0.99, RMSEA 0.01, and SRMR 0.04.

## Discussion

The present study aimed to create and validate a brief version of the IPANAT, a measure for the indirect assessment of affect. Based on the results from the items reduction procedure, three artificial words (i.e., SAFME, TALEP and BELNI) were randomly selected from the six stimuli words used in the IPANAT. Therefore, the brief version of the IPANAT is composed of 18 items. We explored the goodness of fit of IPANAT-18 via CFA technique and found that the best fitting model supports a two-factor structure of the test, corresponding to implicit PA and implicit NA, which is in line with the factor structure found in the original IPANAT (see Quirin et al., 2009). As mentioned in the results section, chi-square and fit indexes indicated a good fit of the proposed model. In addition, the sample size used in the present study was adequate to produce relative stability of the parameter estimates. Internal consistency analyses showed a good reliability for both scales, and the CFA goodness of fit was comparable to findings from previous validations of explicit affect instruments (López et al., 2015).

In our study, PA and NA dimensions occurred to be non-orthogonal, as also reflected in a positive correlation between mean values of implicit PA and implicit NA. This is consistent with previous findings from cross-cultural studies with the IPANAT (see Hernández et al., 2020; Quirin et al., 2018). The authors argued that positive correlations between positive and negative affect could be due the fact that different cultures attribute slightly different meaning to mood adjectives. The latter is also consistent with findings of adjectives referring to personality (Nye et al., 2008). In concordance, previous cross-cultural studies with the IPANAT showed that high correlations between positive and negative affect was mostly due to a positive correlation between the mood adjectives *energetic* and *tense* (Quirin et al., 2018). In addition, it has been argued that in some languages the mood adjectives provide a smaller variability on the responses range. Therefore, future studies exploring this hypothesis should use a sample with a strong emotional context or under emotional priming. Nonetheless, according to Brown (2006) a factor structure with a positive correlation between factors might be the better model fit, particularly if the factor loadings are strong, and the fit indices are better that the one-factor model, as previously found in the IPANAT’s CFAs (see Hernández et al., 2020).

Not least, convergent and discriminant validity of the IPANAT-18 was supported by valence-congruent findings of correlations with explicit affect scales. For example, results showed that correlations between the IPANAT-18 and explicit affect measures are significant and of moderate strength. These moderate correlations are consistent with results reported for the original IPANAT, since Quirin et al. (2009) reported significant correlations of .20 for implicit and explicit PA and .22 for implicit and explicit NA. The moderate correlations between implicit and explicit measures are also consistent with findings of other implicit measures like: the Implicit Association Test (Greenwald et al., 2003), or the Affect Misattribution Procedure (Payne et al., 2005) (See Echebarria-Echabe, 2013). According to some authors, these low correlations between implicit and explicit measures can be due to different aspects, as motivational biases in the explicit measure, lack of introspective access of the participants, or even complete independence of the underlying constructs (Hofmann et al., 2005). In addition, evidence of discriminant validity of the IPANAT-18 can be obtain for our results, since Z-tests showed that implicit NA was more strongly correlated with explicit NA measures than with explicit PA measures, the opposite was found for implicit PA.

Finally, a different set of the artificial words (i.e., SUKOV, GOLIP and KERUS) can be used as a different version of the IPANAT-18. Since results showed that the random selection of items (i.e., three artificial words by 6 mood adjectives) yield similar psychometric properties than the full test. The latter is useful for researchers of the affective phenomena, particularly in experimental settings were repeated measures of the test are needed, since having different version of the test could reduce anchoring effects on participant’s responses.

In conclusion, the present study suggests that the psychometric properties of the IPANAT-18 version are almost as good as those of the full-length measure. Hence, it appears that the shorter measure will serve studies requiring less time for administration than the original test. The latter is especially important for research where affective processes are experimentally induced, since it has been determined that the induced affect is often fleeting (Hermans et al., 2001), so a brief version is useful to better capture these processes. Likewise, research using repeated assessment, as daily-diaries studies, can also benefit by an economical multiple assessment, since a shorter version of instruments will help not to frustrate participants.
